# Expression of Glutamatergic Signaling in Canine Oral Melanocytic Neoplasms

**DOI:** 10.3390/vetsci12121149

**Published:** 2025-12-02

**Authors:** Alison Masyr, Latticha Pluemhathaikij, Sichao Wang, Tuddow Thaiwong-Nebelung, Rebecca C. Smedley

**Affiliations:** 1 Department of Small Animal Clinical Sciences, College of Veterinary Medicine, Michigan State University, East Lansing, MI 48824, USA; 2 Veterinary Diagnostic Laboratory, College of Veterinary Medicine, Michigan State University, Lansing, MI 48910, USA; pluemhat@msu.edu (L.P.); thaiwong@msu.edu (T.T.-N.); smedley1@msu.edu (R.C.S.); 3 Center for Statistical Training and Consulting, Michigan State University, East Lansing, MI 48824, USA; wangsic1@msu.edu

**Keywords:** glutaminase, metabotropic glutamate, melanoma, receptors, dogs

## Abstract

Glutamine addiction in human melanoma is a premier example of the cancer hallmark of metabolic reprogramming. In the present study, we investigate the presence of metabotropic glutamate receptor 1 (mGluR1/*GRM1*) and glutaminase (GLS1/*GLS*) in canine oral malignant melanoma (OMM) and those of low malignant potential, termed histologically well-differentiated melanocytic neoplasm of the lips and oral mucosa (HWDMN). We used immunohistochemistry (IHC) and qPCR to evaluate mGluR1 and GLS1 protein expression and RNA expression, respectively. Nearly 20% of OMM cases had an mGluR1 IHC score ≥ 1, while none of the HWDMN cases had any expression. Due to low IHC expression, only 10 cases were selected for determination of *GRM1* RNA expression, and none were positive. *GLS* RNA expression did not differ between OMM and HWDMN. A GLS1 IHC score ≥ 1 was significantly higher in OMM cases and highly specific (95%) for correctly identifying tumors with a Ki67 index ≥ 19.5. These results may have been negatively impacted by use of a brown chromogen for IHC labeling among background pigment, particularly in HWDMN. Ultimately, these findings suggest that canine OMM does not heavily rely on mGluR1 for tumorigenesis or progression. Differential GLS1 protein expression warrants further investigation with protein quantification.

## Simple Summary

In dogs and people, melanoma is highly locally invasive, metastatic, and chemoresistant. There is a growing body of work in human medicine centered on exploiting aberrant glutamine metabolism vital to melanoma cell survival. In this study, we evaluate the presence of major components of glutamine metabolism in canine oral malignant melanoma (OMM) and histologically well-differentiated melanocytic neoplasm of the lips and oral mucosa (HWDMN), also known as melanocytic tumors of low malignant potential: metabotropic glutamate receptor 1 (mGluR1/*GRM1*) and glutaminase (GLS1/*GLS*). We evaluated immunohistochemical expression of both markers and found that mGluR1 is expressed in less than 20% of OMMs and 0% of HWDMNs. Due to low immunoexpression, only 10 cases were selected for *GRM1* RNA expression evaluation, and none were positive. GLS1 protein immunoexpression was significantly higher in OMMs as compared to HWDMN. On the other hand, RNA expression of *GLS* did not differ between groups. Overall, these results suggest that canine OMM does not heavily rely on mGluR1 for tumor development or progression. Differing GLS1 protein expression warrants further investigation.

## 1. Introduction

Mounting evidence over the last decade has promoted “reprogramming energy metabolism” from classification as an emerging hallmark of cancer to a core hallmark [1]. The most well-documented example of metabolic dysregulation is aerobic glycolysis or the Warburg effect [2]. Glutaminolysis, intracellular conversion of glutamine to glutamate by glutaminase (GLS1), is a less recognized component of the Warburg effect [3]. Glutamine is a critical source of “currency” to cancer cells. As an anaplerotic resource, glutamine can be shuttled into numerous biosynthetic pathways including nucleotide synthesis, glutathione synthesis, and the tricarboxylic acid (TCA) cycle. Cancer cells can become so reliant on glutamine that they experience “glutamine addiction,” whereby lack of this normally non-essential amino acid can induce cellular senescence and death [4,5,6]. In addition to its intracellular value, glutamate has physiologic and pathologic extracellular signaling properties. In physiologic settings, glutamate is an excitatory neurotransmitter. In pathologic settings, glutamate can be used to promote cancer progression, invasion, and metastasis [7,8,9,10].

GLS1 serves as a rate-limiting step in glutamine conversion and subsequent engagement in anaplerosis [11]. Since downstream signaling pathways contribute to cellular proliferation, growth, and survival, GLS1 represents an important component of tumorigenesis. GLS1 is overexpressed in several human cancers, as well as canine mammary tumors [12,13]. Furthermore, GLS1 expression has been correlated with tumor grade in both species [13,14]. Another component of glutamate metabolism utilized by neoplastic cells is metabotropic glutamate receptor 1 (mGluR1). A G-coupled protein receptor, mGluR1 binds glutamate extracellularly and, like GLS1, has been implicated in tumorigenesis [15,16,17,18,19].

Numerous studies have highlighted the role of glutamate metabolism in malignant transformation and progression [12,15,16,17,18,20,21,22]. Specifically, the most pivotal investigations have focused on human melanoma tumorigenesis [16,17,19,23,24,25,26]. Not only do melanoma cells express GLS1 and mGluR1, but they also actively secrete glutamate, unlike normal melanocytes [16]. Individual inhibition of GLS1 and mGluR1 suppresses melanoma cellular proliferation [9,16,17,19,26,27,28]. The impact of glutamine metabolism-related alterations on tumor development and success support that melanomas in humans benefit from the existence of a positive autocrine/paracrine feedback loop. As melanoma develops glutamine addiction, inhibitory therapies can be employed to exploit this vulnerability. Commercially available oral medications, like riluzole and CB-839, can be used to inhibit mGluR1 and GLS1, respectively [9,26,28,29,30,31].

The behavior of canine oral malignant melanoma (OMM) parallels that of human melanoma, specifically the mucosal and acral/digital forms of the human disease [32,33]. Genomic analysis of human mucosal and acral melanoma has not yet centered around mGluR1 or GLS1. However, both forms are known to harbor mutations that converge on major signaling cascades for which glutamine metabolism is integral, namely MAPK and PI3K/AKT [33,34,35,36,37]. Similarly, these two signaling pathways are highly active in canine malignant melanoma [38,39].

Thus, it stands to reason that the demonstrated expression of mGluR1 and GLS1 in human melanoma may carry over to canine OMM. Aberrant expression of glutamatergic metabolic components could hold prognostic and/or therapeutic value in the management of canine oral melanocytic neoplasia. In the current study, we investigate the frequency and extent of mGluR1 and GLS1 expression in spontaneously arising canine OMM and histologically well-differentiated melanocytic neoplasms of the lips and oral mucosa (HWDMN), also known as oral melanocytic neoplasms of low malignant potential. We hypothesize that mGluR1 and GLS1 protein immunoexpression and respective *GRM1* and *GLS* RNA expression, will be high in OMM and negligible in HWDMN. Furthermore, we evaluate this expression within the context of several other established histopathologic prognostic factors for canine oral melanocytic neoplasms.

## 2. Materials and Methods

### 2.1. Sample Selection and Histological Examination

Two groups, comprising OMM and HWDMN, were selected from the Michigan State University Veterinary Diagnostic Laboratory (MSU VDL) archives of formalin-fixed, paraffin-embedded (FFPE) tissues submitted for routine surgical biopsy. Samples were excluded if the tumors had been previously resected or were from metastatic sites. All specimens had been fixed in 10% neutral buffered formalin, routinely processed, embedded in paraffin wax, sectioned at 5 μm, and stained with hematoxylin and eosin (H&E) [40]. Final diagnoses were independently established through histopathologic review by a board-certified veterinary pathologist (RCS) and one anatomic pathology resident (LP). In order to be included, samples had to have 100% diagnostic agreement.

Ki67 index was used as the gold standard for categorizing tumors [40,41]. Inclusion as an OMM was dependent on a Ki67 index ≥ 19.5 and inclusion as an HWDMN was dependent on a Ki67 index < 19.5. HWDMNs also had to exhibit other histologic features of this tumor type, such as high pigmentation, low mitotic count, low nuclear atypia, generally small (<1 cm), often raised, non-ulcerated, and generally containing abundant collagenous stroma [40,41,42]. Cases were preferentially selected if immunolabeling for Ki67 had been performed previously. If previously labelled Ki67 index was repeated to confirm accuracy. Immunolabeling for Ki67 and determination of a Ki67 index were performed for any tumor that had not been previously evaluated for Ki67.

### 2.2. Immunohistochemistry and Immunohistochemical Examination

Immunohistochemical (IHC) labeling for mGluR1 and glutaminase GLS was performed on all cases in both the OMM and HWDMN groups (RCS and LP). Appropriate positive and negative controls were used based on manufacturer’s recommendation.

For mGluR1 (clone D5H10; Cell Signaling Technology, catalog #12551S, Danvers, MA, USA), 5 μm FFPE sections were dewaxed and subjected to high pH heat-induced epitope retrieval (HIER) using the DAKO PT Link system. Staining was conducted on the Dako Autostainer Link 48 platform using the EnVision FLEX+ high pH detection system (Agilent, catalog #K800221-5, Santa Clara, CA, USA) with 3,3′-diaminobenzidine (DAB) as the chromogen. The antibody was applied at a dilution of 1:100 and incubated at room temperature for 30 min. Since mGluR1 is expressed in human brain tissue [7], we tested canine brain tissue and identified canine pituitary gland served as a positive control, and canine brainstem as a negative control (Figure S1).

For GLS (MyBioSource, catalog #MBS9612093, San Diego, CA, USA), 5 μm sections were dewaxed using the Roche HE600 system, and staining was performed without antigen retrieval. The antibody was applied at a 1:500 dilution and incubated at room temperature for 30 min. The same Dako Autostainer Link 48 platform and EnVision FLEX+ detection system were used. Given that GLS is expressed in human and rat renal tissue [43,44], we tested canine renal tissue and identified renal tubular epithelial cells as a positive control, and renal glomeruli and blood vessels as negative controls (Figure S2).

For cases that had not been previously immunolabeled for Ki67, IHC labeling for Ki67 was performed according to the methods of Bergin et al. [41] Briefly, Ki67 immunolabeling was performed on the Benchmark Automated Staining System (Ventana Medical Systems, Inc., Tucson, AZ, USA) following heat-induced epitope retrieval. A mouse monoclonal anti-Ki67 antibody (MIB-1; Dako Cytomation, Carpinteria, CA, USA) at dilution 1:50 was applied and detected using the Enhanced V-Red detection system (Ventana Medical Systems, Inc., Tucson, AZ, USA), which used alkaline phosphatase and the chromogen AEC. Positive and negative controls were used [41].

For cases requiring confirmation of melanocytic origin, IHC was performed using the MDX cocktail (Melan-A, PNL2, TRP-1, and TRP-2) on the Dako Omnis platform with the EnVision FLEX HRP Magenta detection system (Agilent, Santa Clara, CA, USA). Any tumors that did not meet these criteria were excluded. Positive and negative controls were included in all runs for each antibody [45,46].

IHC-labeled slides were semi-quantitatively scored by one board-certified veterinary pathologist (RCS) and one anatomic pathology resident (LP). The percentage of positively labeled neoplastic cells was scored as follows: ≤10% (score 0), 11–25% (score 1), 26–50% (score 2), 51–75% (score 3), and >75% (score 4) [46].

For MDX, tumors were considered positive if >10% of the neoplastic cells labeled, if intraepithelial nests consisting of ≥5 contiguous neoplastic cells demonstrated labeling, or if clusters of ≥20 positive neoplastic cells were identified anywhere within the tumor.

### 2.3. RNA Extraction and Reverse Transcriptase Digital Polymerase Chain Reaction (qPCR)

*GRM1* and *GLS* copy number were assessed by exploiting an absolute Q Digital-PCR (dPCR)-System (Applied Biosystems, Waltham, MA, USA). RNA in FFPE tissue from dogs with OMM and HWDMN were extracted using RecoverAll™ Total Nucleic Acid Isolation Kit for FFPE (Thermo Fisher Scientific, Waltham, MA, USA). Purified RNAs (2 ng) were reverse-transcribed and treated with ezDNAse using a SuperScript IV VILO (SSIV VILO) Master Mix kit (Thermo Fisher Scientific, Waltham, MA, USA). Reactions were set up according to the manufacturer’s instructions. cDNA was added in the PCR reactions (Absolute Q DNA Digital PCR Master Mix Thermo Fisher Scientific, Waltham, MA, USA) and 20x Taqman^TM^ Gene Expression assay (Assay ID for *GLS*: Cf01014021_m1, Thermo Fisher Scientific, Waltham, MA, USA; Assay ID for *GRM1*: ARRWJEU, Thermo Fisher Scientific, Waltham, MA, USA) in a total of 10 μL. Next, 9 μL of the reaction mix was loaded to QuantStudio MAP16 Digital PCR Plate with 15 μL of QuantStudio Absolute Q Isolation Buffer overlay. The reaction was run in the thermal cycler (QuantStudio Absolute Q Digital PCR System, Thermo Fisher Scientific, Waltham, MA, USA) under the following conditions: 10 min at 96 °C, and 40 cycles of 96 °C for 5 s and 60 °C for 15 s. No RT negative control was also included. Partitions were analyzed for fluorescent measurement of fluorescein amidite (FAM) probe with QuantStudio Absolute Q Analysis Software version 1.0.46.13 (Thermo Fisher Scientific, Waltham, MA, USA). *GAPDH* copy number was also quantified in separate reactions and used as a reference in data analysis [47]. In brief, based on the theoretical amount of RNA used as input (2 ng) and the obtained number of *GAPDH* copies/reaction, the number of *GRM1* or *GLS* copies per ng input RNA was finally inferred.

### 2.4. Data Analysis

All analyses were performed using R (version 4.5.0; R Core Team, 2025) with package pROC. Categorical variables were summarized using frequencies and percentages. Continuous variables were reported as means ± standard deviations or medians with interquartile ranges (IQR), as appropriate to distribution. Fisher’s exact test was used for GLS1 IHC score, and Wilcoxon rank-sum test was used for *GLS* RNA expression. Prognostic performance was evaluated with ROC curves for GLS1 IHC score and *GLS* RNA expression against Ki67 ≥ 19.5 as the gold standard. Significance was set at *p* < 0.05.

## 3. Results

### 3.1. Case Selection and Histologic Assessment

Fifty-three cases of canine oral melanocytic neoplasms were selected from the MSU VDL. The OMM group included 34 dogs with tumors characterized by variably pleomorphic, variably pigmented neoplastic cells that generally had high mitotic counts (>3 mitoses in 2.37 mm^2^ and nuclear atypia (≥30% nuclear atypia) and sometimes demonstrated junctional activity. The HWDMN group included 19 dogs with tumors composed of well-differentiated, heavily pigmented melanocytes with generally low mitotic activity and nuclear atypia. A summary of common histopathologic prognostic factors is shown in Table 1.

### 3.2. Protein Expression

**Figure 1:**
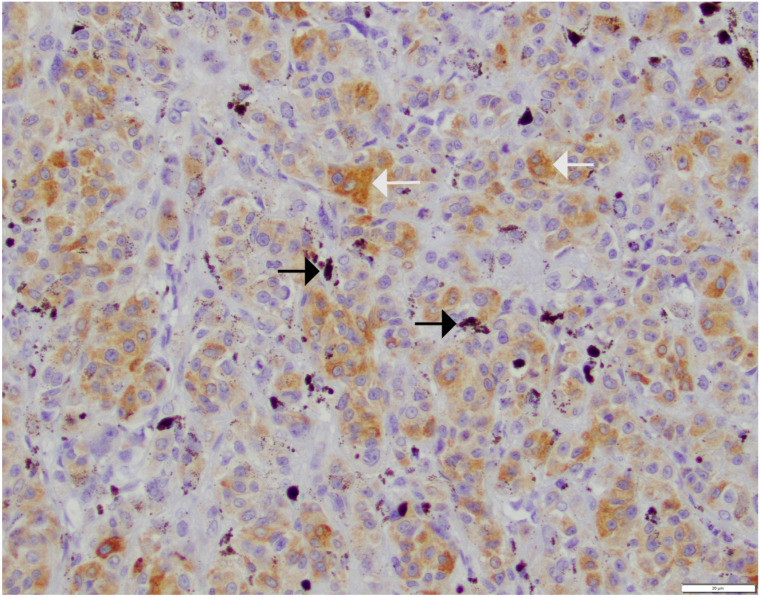
Canine oral malignant melanoma with an area of immunohistochemical brown labeling for mGluR1 at a score of 1 (10–20% of tumor cells positive). Note that melanin (dark brown pigment—black arrows) is distinguishable from the brown chromogen (orange to light brown—white arrows). Scale bar in lower right corner is equivalent to 20 μm.

Protein expression for mGluR1 and GLS1 was performed via IHC labeling. Among OMM cases, 28/34 (82%) had a mGluR1 IHC score of 0. The remaining 6 cases scored as follows: 1 case scored 1 (Figure 1), 1 case scored 2, 1 case scored 3, and 3 cases scored 4. Among HWDMN cases all 19 cases scored 0 for mGluR1 expression on IHC.

**Figure 2:**
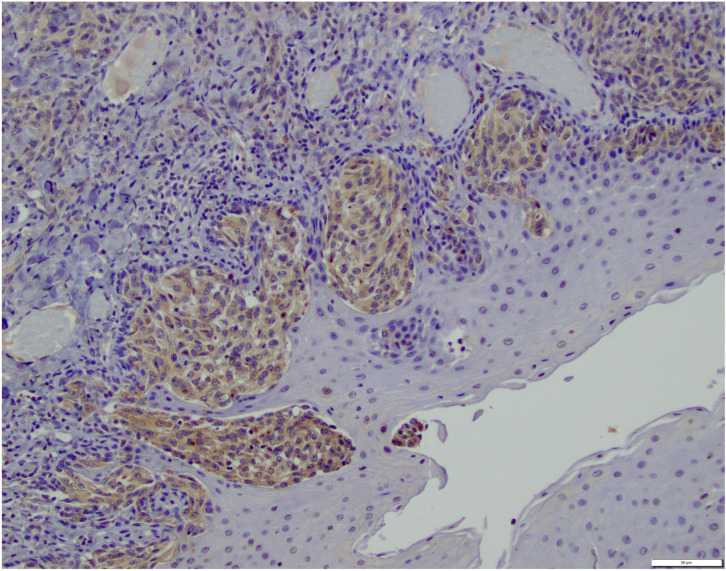
Canine oral malignant melanoma with immunohistochemical brown labeling for GLS1 at a score of 2 (40% of tumor cells positive). Scale bar in lower right corner is equivalent to 20 μm.

**Figure 3:**
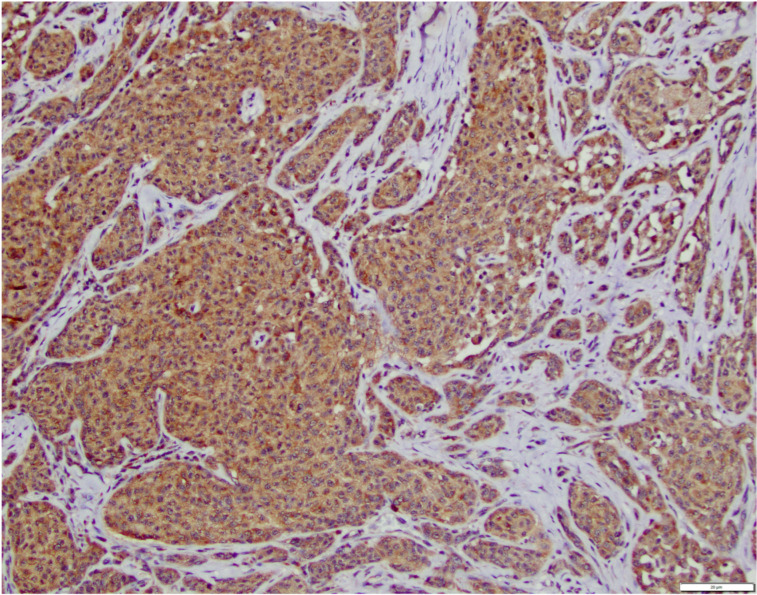
Canine oral malignant melanoma with immunohistochemical brown labeling for GLS1 at a score of 4 (90% of tumor cells positive). Scale bar in lower right corner is equivalent to 20 μm.

GLS1 IHC scoring revealed that 10/34 (29%) of OMM cases scored 0 (Table 2). The remaining 24 cases scored as follows: 3 cases scored 1, 2 cases scored 2 (Figure 2), 5 cases scored 3, and 14 cases scored 4 (Figure 3). Among HWDMN cases, 18/19 (95%) scored 0. One case was scored 3.

### 3.3. RNA Expression

Given the low frequency and intensity of mGluR1 scoring, *GRM1* qPCR was only performed on 10 cases as a test: 8 OMM and 2 HWDMN. Three of the OMM cases had an IHC score of 0, the remaining 5 were score 1 or higher. All 10 cases were found to have no quantifiable *GRM1* RNA expression (less than 0 copies/ng RNA). Therefore, *GRM1* qPCR was not performed on the remaining tumors.

Overall median *GLS* RNA expression was 223.7 copies/ng RNA from 49 cases. Four HWDMN cases did not have enough RNA for evaluation. Median *GLS* RNA expression was 244 copies/ng RNA for OMM cases and 156 copies/ng RNA for HWDMN cases.

### 3.4. Statistical Analysis

GLS1 IHC score differed significantly between OMM and HWDMN cases (*p* < 0.001). Furthermore, GLS1 IHC score was highly correlated with Ki67 index and mitotic count (Spearman’s ρ = 0.68 and 0.60, respectively). Additionally, pigmentation of <50% and nuclear atypia of ≥30% were moderately correlated with GLS1 IHC score (Spearman’s ρ = 0.58 and 0.43, respectively). *GLS* RNA expression did not differ between OMM and HWDMN (*p* = 0.2). Correlation coefficients between *GLS* RNA expression and Ki67 index, mitotic count, pigmentation, and nuclear atypia were all low (Spearman’s ρ < 0.26). Similarly, there was a low correlation between GLS1 IHC score and *GLS* RNA expression (Spearman’s ρ = 0.26).

Using the Ki67 index as the gold standard to differentiate OMMs from HWDMN tumors, ROC curve analysis showed area under the curve for GLS1 IHC score and *GLS* RNA expression was 0.83 and 0.62, respectively. GLS1 IHC score ≥ 1 had the greatest accuracy of predicting a tumor Ki67 index ≥ 19.5. Accuracy was 0.79 with sensitivity of 0.71, specificity of 0.95, positive predictive value of 0.96, and negative predictive value of 0.64 at the study prevalence of Ki67 index ≥ 19.5 of 64.2% (34/53).

*GLS* RNA ≥ 164 copies/ng RNA had the greatest accuracy of predicting a tumor Ki67 index ≥ 19.5. Accuracy was 0.65 with sensitivity of 0.68, specificity of 0.60, positive predictive value of 0.79, and negative predictive value of 0.45 at the study prevalence of Ki67 index ≥ 19.5 of 64.2% (34/53).

Given the limited findings regarding mGluR1 IHC score and *GRM1* RNA expression, minimal statistical analysis was performed with these variables.

## 4. Discussion

Glutaminase protein expression differed significantly between OMM and HWDMN, which is in line with previous literature on human cutaneous melanoma compared to normal melanocytes [27]. Conversely, *GLS* RNA expression did not differ between the two groups. Our preliminary results also showed that there was minimal mGluR1/*GRM1* protein expression in canine oral melanocytic tumors, regardless of malignancy status. These results are in contrast with previous publications on human cutaneous melanoma compared to normal melanocytes [16,17]. The lack of mGluR1 RNA quantification in assessed samples could raise concern for primer accuracy and this is countered by the positive control tissue used yielding results.

Immunohistochemical examination determined that GLS1 expression was significantly different between highly aggressive OMM tumors and HWDMN of low malignant potential. This finding warrants further exploration with western blot assessment and protein quantification. Future investigations would ideally include fresh tissue and compare OMM, HWDMN, digital melanoma, cutaneous melanocytoma, cutaneous malignant melanoma, and normal melanocytes.

Glutaminase protein expression can be affected by a host of cellular factors. Since the RNA transcript level did not differ between OMM and HWDMN samples, we have considered if protein degradation mechanisms could vary between well-differentiated and malignant melanocytic tumors. GLS1 experiences diphenylarsinic acid (DPAA)-mediated conformational change, which is believed to trigger breakdown via mitochondrial Lon protease [48]. If intracellular DPAA or Lon protease levels are decreased in OMM, this would lead to prolonged GLS1 presence. We did not evaluate DPAA or Lon protease gene or protein expression as part of this study. A growing body of work surrounding Lon protease expression in cancer suggests that Lon protease is often overexpressed as a component of metabolic reprogramming, a hallmark of cancer [49,50,51,52]. As such, it is improbable that canine OMM would be unique in relying on decreased Lon expression to facilitate prolonged GLS1 cellular presence. Indeed, post-translational modifications and antibody specificity could have contributed to the discordance between IHC and qPCR results.

Approximately 18% of OMM samples had an mGluR1 IHC score ≥ 1 and it is interesting to note that none of the 19 HWDMN samples were positive. Similarly, 71% of OMM samples were positive for GLS1 via IHC, while only 5% of HWDMN cases were positive. While this reveals mGluR1 and GLS1 IHC to be of low sensitivity for differentiating between OMM and HWDMN, they may prove to have more specificity. Given the limited numbers of positive samples for mGluR1 IHC, in-depth statistical analysis was not explored for this marker, although the high specificity and positive predictive value of GLS1 IHC score ≥ 1 in identifying tumors with a Ki67 index ≥ 19.5 support its value as a prognostic marker. Ultimately, the present data is not strong enough to warrant relying on GLS1 IHC score alone, and it is unlikely to prove superior in differentiating highly aggressive and less aggressive oral tumors as compared to other known markers.

Given the incongruency between the GLS1 IHC and *GLS* RNA quantification results, it is also possible that the former were subject to false negative interpretation. The IHC protocol employed used a brown chromogen. Distinguishing the brown chromogen from melanin, particularly in highly pigmented HWDMN, can be done, but it presents a challenge and can create an opportunity for false negative results. To mitigate this, our laboratory attempted protocols using bleaching and labeling with a red chromogen, although neither approach allowed for definitive interpretation, and they were not pursued further. None of the data presented in this manuscript was subjected to bleaching or labeling with a red chromogen. Future studies could more confidently confirm the presence of GLS1 by validating an IHC protocol with bleaching and/or labeling with a red chromogen.

It is also important to note that we did not compare protein or RNA expression of GLS1 and mGluR1 between OMM and to normal non-neoplastic melanocytes, as these cells are typically dispersed throughout normal tissue. It is possible that our findings regarding IHC and RNA expression in both OMM and HWDMN are relatively high as compared to normal melanocytes. Either mGluR1 and/or GLS1 could be implicated in canine melanocytic tumorigenesis, although the extent may not differ between these two forms of disease. Moreover, the absence of strong correlations limits translational or prognostic conclusions. We did not include normal melanocyte samples in this study as identifying large aggregates of normal melanocytes is challenging. Future studies comparing OMM to cutaneous melanocytomas, melanocytic tumors typically of especially low malignant potential, may help to further determine if GLS1 and/or mGluR1 expression is relatively higher in oral melanocytic tumors.

Glutamine addiction, facilitated by mGluR1 and GLS1, is a well-documented phenomenon in human cutaneous melanoma [11,16,17,18,27,53]. The current literature centers mostly on cutaneous melanoma, although canine OMM appears to more closely reflect the behavior of mucosal and acral/digit melanoma in people [32,33]. All of the canine and human forms of melanoma mentioned rely on the downstream MAPK and PI3K/AKT signaling pathways [34,35,36,37,38,39]. It is important to keep in mind that mGluR1 and GLS1 do not represent the entirety of glutamate metabolic cellular machinery. Other components of this metabolic cascade may play a role in tumor development and progression. However, the findings of the current study suggest that canine OMM does not heavily rely on mGluR1 for tumorigenesis or progression. Our IHC findings for GLS1 warrant further study and may yield future therapeutic options. As energy reprogramming is a hallmark of cancer, it is possible that OMM utilizes other aberrant metabolic pathways that could become the center of future investigations.

## Figures and Tables

**Table 1:** Summary of Histopathologic Prognostic Factors.

Characteristic	OMM	HWDMN
Pigmentation < 50%	29/34 (85%)	0/19 (0%)
Nuclear Atypia ≥ 30%	31/34 (91%)	5/19 (26%)
Ki67, ≥19.5	34/34 (100%)	0/19 (0%)
Ki67, Median (Range)	78.3 (23–376)	3.0 (0–16.2)
Mitotic Count > 3/2.37 mm^2^	33/34 (97%)	3/19 (16%)
Mitotic Count/2.37 mm^2^, Median (Range)	31 (2–127)	1 (0–8)

**Table 2:** Glutaminase Immunohistochemical Scoring.

Score	OMM	HWDMN
0	10 (29%)	18 (95%)
1	3 (9%)	0 (0%)
2	2 (6%)	0 (0%)
3	5 (15%)	1 (5%)
4	14 (41%)	0 (0%)

## Data Availability

The original contributions presented in this study are included in the article. Further inquiries can be directed to the corresponding author.

## Supplementary Materials

The following supporting information can be downloaded at https://www.mdpi.com/article/10.3390/vetsci12121149/s1, Figure S1: Canine brain tissue demonstrating positive mGluR1 labeling in pituitary (A) and negative mGluR1 labeling in brainstem (B). Figure S2: Canine renal tissue demonstrating positive GLS1 labeling in tubular epithelial cells and negative GLS1 labeling in glomeruli and blood vessels.
