# Supplementary material for: Expression of Glutamatergic Signaling in Canine Oral Melanocytic Neoplasms
Source: Vet Sci. 2025 Dec 2;12(12):1149. doi: 10.3390/vetsci12121149 (PMC12737758; doi:10.3390/vetsci12121149)
Supplement: Supplementary file 1 [file vetsci-12-01149-s001.zip › vetsci-3917694-Supplementary Materials.pdf]

Supplementary Materials

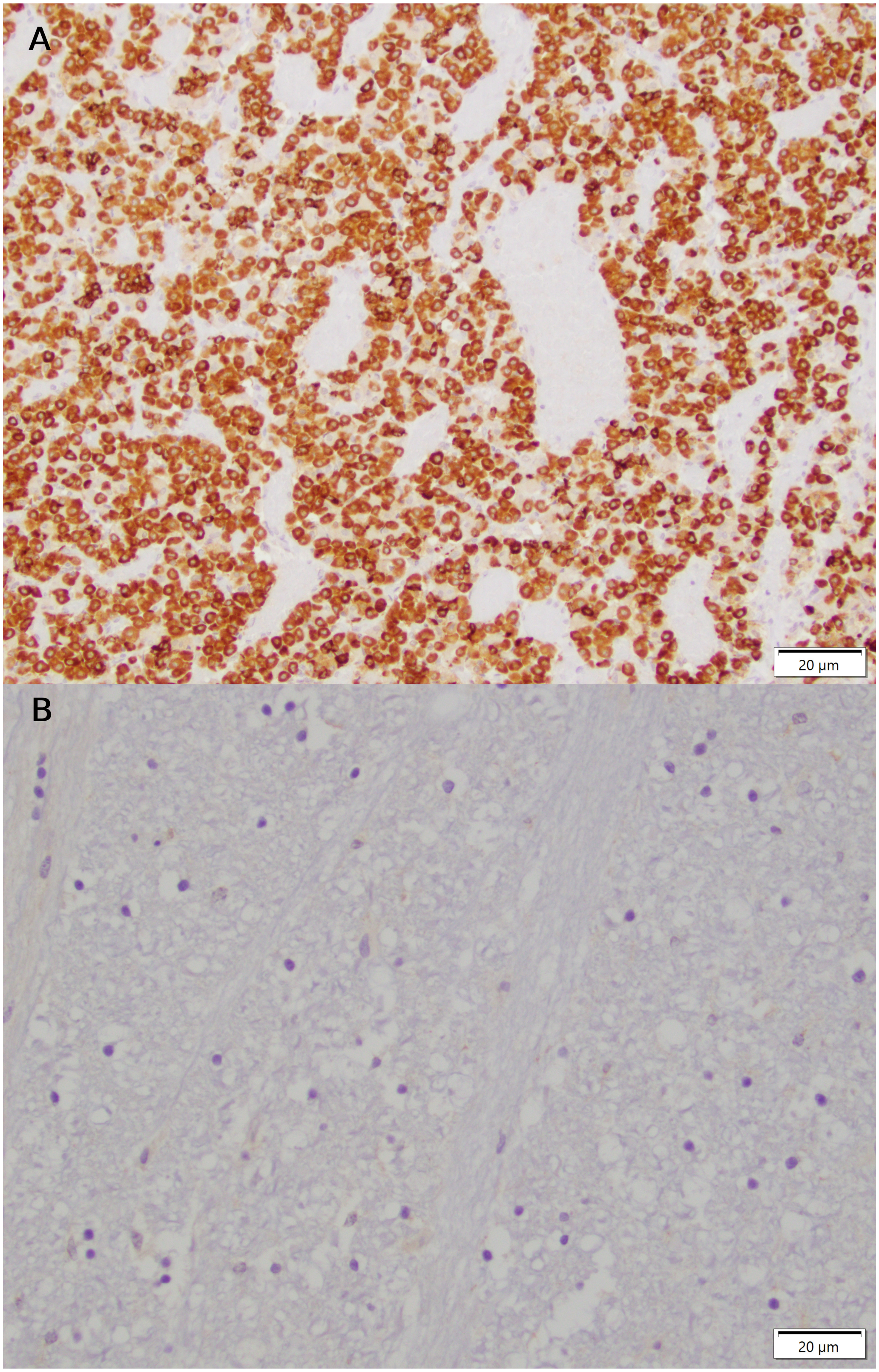

Figure S1. Canine brain tissue demonstrating positive mGluR1 staining in pituitary (A) and negative mGluR1 staining in brainstem (B).

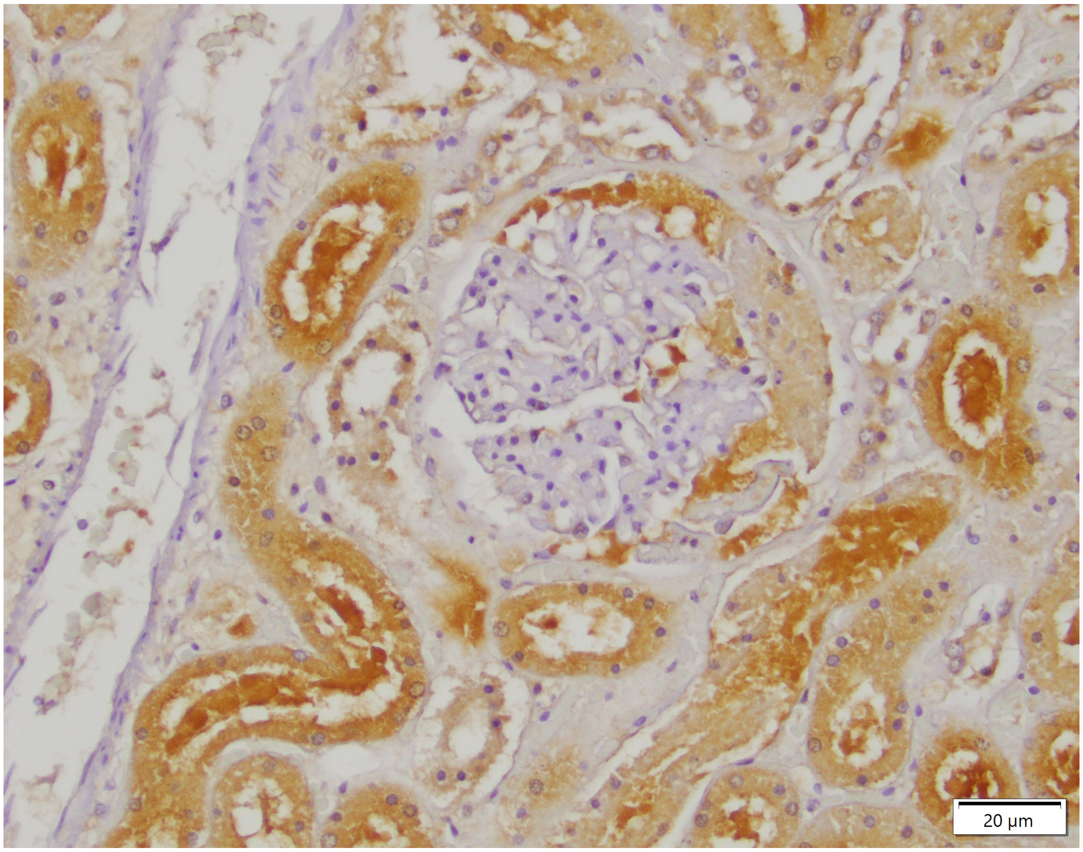

Figure S2. Canine renal tissue demonstrating positive GLS1 staining in tubular epithelial cells and negative GLS1 staining in glomeruli and blood vessels.
